# Implementing a Health Utility Assessment Platform to Acquire Health Utilities in a Hemodialysis Outpatient Setting: Feasibility Study

**DOI:** 10.2196/33562

**Published:** 2022-07-28

**Authors:** Adeboye A Adejare, Heather J Duncan, R Geoffrey Motz, Silvi Shah, Charuhas V Thakar, Mark H Eckman

**Affiliations:** 1 Department of Biomedical Informatics University of Cincinnati Cincinnati, OH United States; 2 Division of Nephrology and Hypertension University of Cincinnati Cincinnati, OH United States; 3 Division of General Internal Medicine and the Center for Clinical Effectiveness University of Cincinnati Cincinnati, OH United States

**Keywords:** health utility assessment, patient reported outcomes, end-stage kidney disease, hemodialysis, hepatitis C

## Abstract

**Background:**

Patients with end-stage kidney disease (ESKD) wait roughly 4 years for a kidney transplant. A potential way to reduce wait times is using hepatitis C virus (HCV)–viremic kidneys.

**Objective:**

As preparation for developing a shared decision-making tool to assist patients with ESKD with the decision to accept an HCV-viremic kidney transplant, our initial goal was to assess the feasibility of using The Gambler II, a health utility assessment tool, in an ambulatory dialysis clinic setting. Our secondary goals were to collect health utilities for patients with ESKD and to explore whether the use of race-matched versus race-mismatched exemplars impacted the knowledge gained during the assessment process.

**Methods:**

We used The Gambler II to elicit utilities for the following ESKD-related health states: hemodialysis, kidney transplant with HCV-unexposed kidney, and transplantation with HCV-viremic kidney. We created race exemplar video clips describing these health states and randomly assigned patients into the race-matched or race-mismatched video arms. We obtained utilities for these 3 health states from each patient, and we evaluated knowledge about ESKD and HCV-associated health conditions with pre- and postintervention knowledge assessments.

**Results:**

A total of 63 patients with hemodialysis from 4 outpatient Dialysis Center Inc sites completed the study. Mean adjusted standard gamble utilities for hemodialysis, transplant with HCV-unexposed kidney, and transplantation with HCV-viremic kidney were 82.5, 89, and 75.5, respectively. General group knowledge assessment scores improved by 10 points (*P*<.05) following utility assessment process. The use of race-matched exemplars had little effect on the results of the knowledge assessment of patients.

**Conclusions:**

Using The Gambler II to collect utilities for patients with ESKD in an ambulatory dialysis clinic setting proved feasible. In addition, educational information about health states provided as part of the utility assessment process tool improved patients’ knowledge and understanding about ESKD-related health states and implications of organ transplantation with HCV-viremic kidneys. A wide variation in patient health state utilities reinforces the importance of incorporating patients’ preferences into decisions regarding use of HCV-viremic kidneys for transplantation.

## Introduction

Chronic kidney disease disproportionally impacts African Americans, making it a prototypical disease in which to investigate disparities in health utility assessments (HUAs) [1]. Due to the limited availability of organs and the large number patients with end-stage kidney disease (ESKD) on waiting lists for transplantation, the average patient waits roughly 4 years before receiving a kidney transplant [2]. Patients with African American racial background who have ESKD wait even longer at an average of 4.5 years [2-4]. One path toward increasing the availability of organs and reducing waiting times is to use hepatitis C virus (HCV)–viremic kidneys for transplantation. The decision to accept transplantation with an HCV-viremic kidney hinges on the balance between the decreased waiting time afforded by accepting such an organ and each patient’s values and preferences about receiving such an organ. These trade-offs make this decision an ideal setting for shared decision-making (SDM) [5]. SDM clarifies patients’ values and preferences, can improve self-efficacy, and engages patients in conversations with their clinicians about treatment choices [5,6].

As of 2017, a total of 746,557 patients in the United States have ESKD [3]. A total of 101,337 patients with ESKD were wait-listed for kidney transplantation in 2019, while the number of patients receiving kidney transplants in 2019 was 24,273 [7]. Studies show that receiving a kidney transplant, even with an HCV-viremic organ, can improve the survival and quality of life and reduce lifetime costs for patients with ESKD who are currently on dialysis [8]. Importantly, using HCV-viremic organs can increase the availability of otherwise high-quality organs and potentially reduce the waiting time for some patients to receive kidney transplants [9]. However, some patients, even after undergoing successful transplantation with an HCV-viremic kidney, have worries and concerns that continue to impact their quality of life. Thus, including individual patient’s values and preferences regarding the relevant health states is a critical component of SDM. In anticipation of the future development of an SDM tool, the primary goal of this study was to assess the feasibility of using The Gambler II, a health utility assessment tool, in an ambulatory dialysis clinic setting. Our secondary goals were to collect utilities for patients with ESKD and to explore whether the use of race-matched versus race-mismatched exemplars impacted the knowledge gained during the assessment process.

The Gambler II is a health utility assessment software platform that can use a variety of assessment techniques to gather patients’ utilities [10,11]. These include the visual analog scale (VAS), standard gamble (SG), and time trade-off (TTO) [12] (Multimedia Appendix 1 A). The utility assessment process itself provides an opportunity to educate patients about the health states for which values and preferences are being sought [13,14]. The Gambler II uses video clips of patient actors to describe health states. In addition, The Gambler II can match the demographics of patient actors in the video clips to those of the patient whose utilities are being assessed. This latter feature provided us with an opportunity to explore a hypothesis, based on exemplification theory, that information communicated by people who mostly resemble the participant is more likely to engage and inform the participant [15,16].

Our longer-term goal is to develop an SDM tool that can be used by clinicians to facilitate discussions about acceptance of HCV-viremic kidneys, particularly for patients who may have longer predicted waiting times on transplant lists. The tool would assess patients’ utilities for hemodialysis and transplantation with either an HCV-unexposed or an HCV-viremic kidney and would use that information along with patient-specific demographic information and predictions of organ availability based on factors including age, sex, blood type, dialysis vintage, calculated panel reactive antibodies, comorbidities, and the region in which the transplant is being carried out to make a recommendation for the best transplantation strategy for that patient. As many patients on dialysis, particularly African Americans living in less affluent urban areas, are impacted by the digital divide and have less access to home computers and the internet [6], we envision using this tool in ambulatory dialysis clinic settings with readily available computing platforms such as laptop and tablet computers.

## Methods

### Ethics Approval

This study was reviewed and approved by the University of Cincinnati Institutional Review Board (UC IRB ID – 2019-0792) as well as by the Dialysis Clinic Inc Administrative Review Office. Both boards approved the study before patient recruitment began. No compensation was provided to the patients for this research.

### Study Design

#### Patient Recruitment

We worked with physicians in the Division of Nephrology and Hypertension to help recruit patients with chronic hemodialysis receiving treatment at any of the 4 outpatient dialysis centers in the greater Cincinnati metropolitan area managed by Dialysis Center Inc (DCI) [17]. Inclusion criteria were a diagnosis of ESKD, receiving intermittent facility hemodialysis at the designated outpatient center (DCI), and the ability to understand the English language. We included adults between the ages of 21 and 80 years. Patients with significant cognitive or reading deficits were excluded from the study. Patients who did not opt out of being contacted were given further explanation on the purpose of the study to assess their interest and willingness to participate.

#### Study Flow

**Figure 1:**
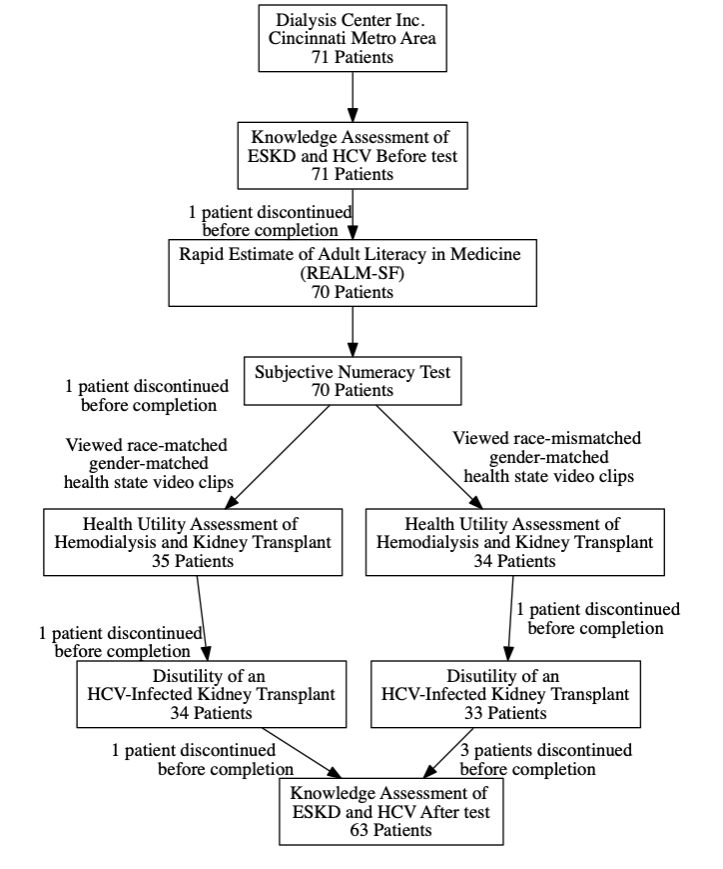
Study flow. ESKD: end-stage kidney disease; HCV: hepatitis C virus.

Patients were recruited from 4 DCI sites in the Cincinnati metropolitan region. Prior to participating in the utility assessment process, they underwent a knowledge assessment survey. We collected demographic information and assessed health literacy using the Rapid Estimate of Adult Literacy in Medicine and subjective numeracy. We randomized patients to 1 of 2 study arms in which they viewed either race-matched or race-mismatched video clips describing each of the 3 health states. The patients’ utilities for these health states were obtained, and then a postinterview knowledge assessment was repeated (Figure 1). Those patients who indicated interest were met by the study principal investigator (AAA) in their local dialysis center, underwent a formal consent process, and answered on a laptop computer a minimal amount of demographic and clinical information, completing a short survey on educational status, time on dialysis, history of prior kidney transplant, and interest in a future kidney transplant. The patients then completed a Research Electronic Data Capture (REDCap) survey evaluating subjective numeracy and health literacy along with a short previsit knowledge assessment [18-20]. At any time, patients could withdraw from the study and did not have to provide a reason for withdrawal. To understand preintervention state of knowledge about hemodialysis, HCV infection, and kidney transplantation, we developed a 10-item multiple-choice questionnaire (Multimedia Appendix 1 B).

**Figure 2:**
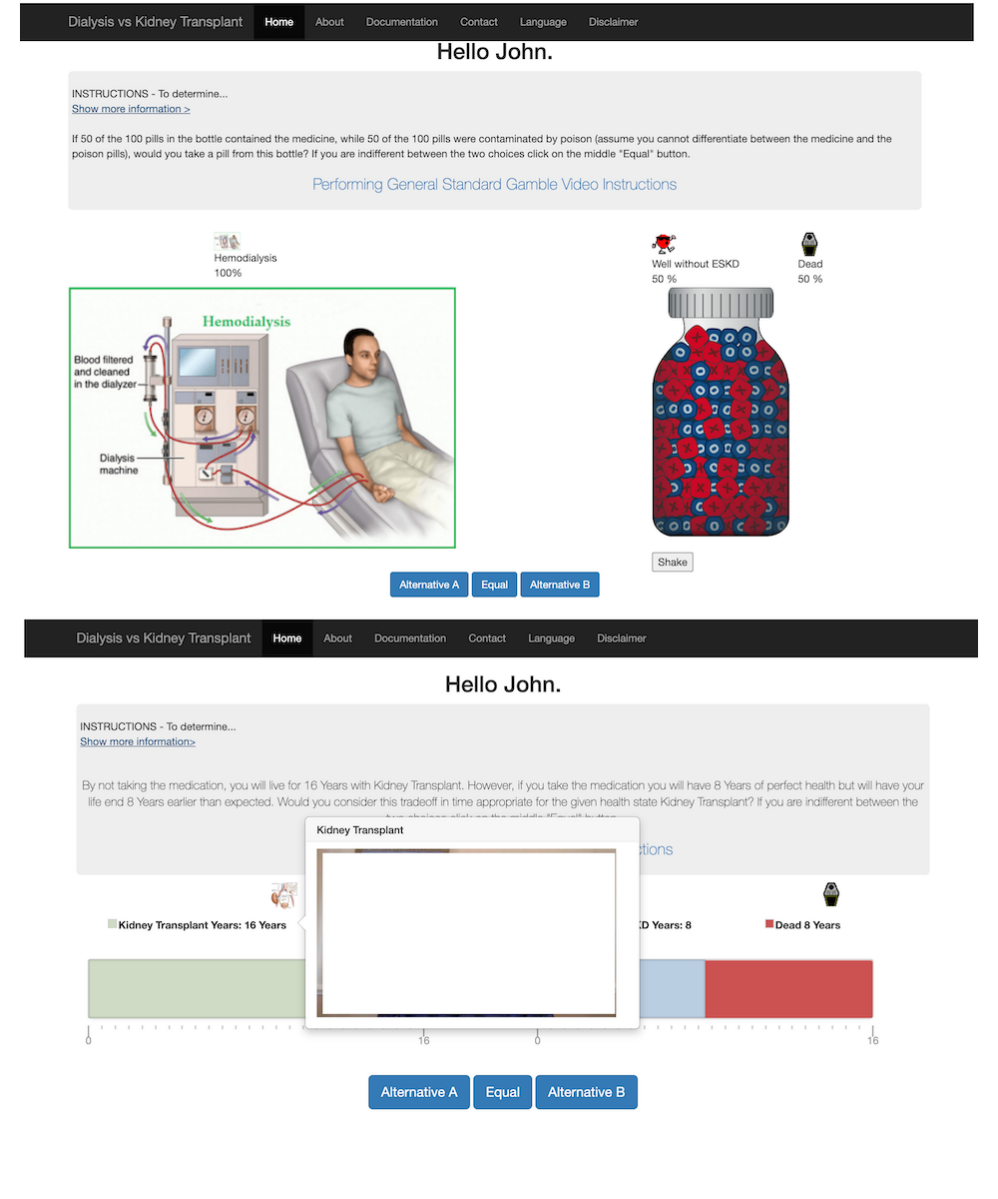
Screenshots of different health utility assessments. Top: patient evaluating the hemodialysis health state through the standard gamble utility assessment. Bottom: patients evaluating kidney transplant using the time trade-off; The Gambler uses life tables to determine the duration of life expectancy for the time trade-off. The video clip in the figure demonstrates how a user can watch a demographically matched patient actor describe the health state being assessed. We did not incorporate the image of the patient actor to protect their privacy. ESKD: end-stage kidney disease.

**Figure 3:**
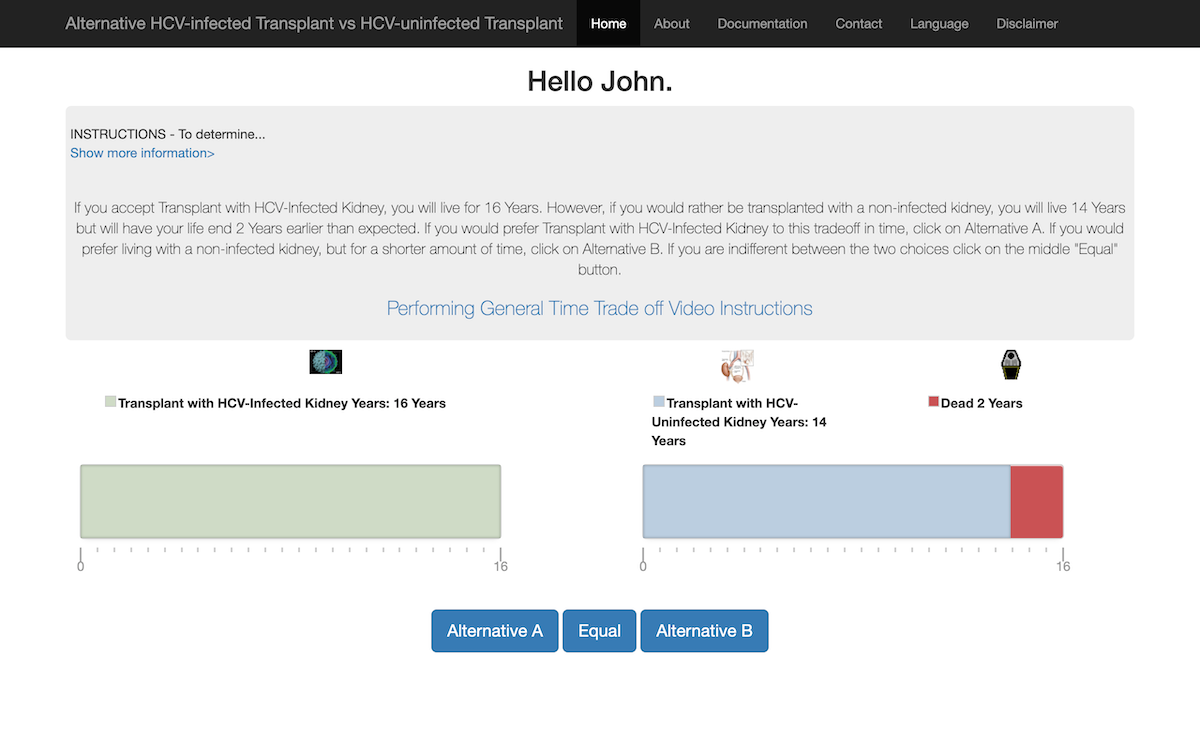
Screenshot of patient going through the time trade-off health utility assessment for transplantation with a hepatitis C virus (HCV)–infected kidney. This assessment differs from others in that the best anchor health state is a transplantation with an uninfected kidney.

One of our secondary goals was to evaluate the impact of patients viewing race-matched versus race-mismatched video clips describing the health states being assessed. As mentioned, exemplification theory suggests that people are more engaged and receptive when they are presented with visual information by people who look most like themselves [16]. We hypothesized that learning about relevant health states would be greater among patients who receive video clip information from patient actors who are similar to them as opposed to receiving information from those who are different (eg, race, gender, and age category). We evaluated this by measuring the change score on the knowledge survey given before and after the HUA sessions. We did not provide any training materials prior to the HUA sessions and did not assume patients had knowledge about hepatitis C or any other knowledge besides their current understanding of dialysis and kidney transplant. We determined that 30 patients would be required in each study arm to detect a difference as small as 12 points (on a scale of 0 to 100) in the change score between pre- and postknowledge survey (power of 0.80 with an alpha of .05), using a 2-sided *t* test. We randomized patients into 1 of 2 study arms (Multimedia Appendix 1 C). The first arm presented patients with video clips of race- and gender-matched health state descriptions, while the second arm presented patients with video clips purposely mismatched for race (Figure 2 and Figure 3). The entire interview process lasted roughly 1 hour and took place while patients were receiving their 3- to 4-hour long dialysis treatment.

Another secondary goal of our study was to collect utilities for the 3 ESKD-related health states described above. While studies have assessed patient utilities for hemodialysis and kidney transplantation, to our knowledge, none have explored health utilities for transplantation with HCV-viremic kidneys [21,22]. We collected these data using the 3 HUA methods of visual analog scale (“feeling thermometer”), standard gamble, and time trade-off. We broke the assessment process into 2 parts. First, we assessed health states of intermittent hemodialysis and transplantation with an HCV-unexposed kidney, using anchor states of “Well” (without ESKD) and “Dead.” We next assessed the health state of transplantation with an HCV-viremic kidney using transplantation with an HCV-unexposed kidney as the best outcome and Dead as anchor states. Patients entered one of these two HUA groups to explicitly assess how much of a risk they were willing to take (in the standard gamble) to avoid receiving an HCV-viremic kidney given the opportunity of a noninfected kidney transplant (Multimedia Appendix 1 D).

To control for possible confounding, we also collected demographic information (age, sex, and race), highest educational level attained, dialysis vintage, history of prior kidney transplant, interest in receiving a transplanted kidney, health literacy using the Rapid Estimate of Adult Literacy in Medicine Short Form, and subjective numeracy [20-22].

### Tools

We conducted surveys and performed HUAs using a laptop computer (2014 Apple MacBook Pro running Mac OS 10.14) while we met with most patients in their community dialysis center during their usual intermittent hemodialysis sessions. Patients used either headphones, earbuds, or audio output from the laptop to hear the video clips that were part of The Gambler II. We used Google Chrome version 80 as the web browser to access The Gambler II and REDCap (9.1.10). Patient actors used a standard script for each health state, which was developed and vetted by team members (MHE, HJD, SS, CVT, and GRM). We recorded 12 different video clips describing the 3 health states for all combinations of gender and race (ie, men, women, African American, and White) using Apple’s QuickTime X 10.5 with video mastering done on Adobe Premiere and Adobe Audition 2019. Due to restrictions of social distancing during the COVID-19 pandemic, the institutional review board granted an amendment to the protocol that allowed us to interview some patients remotely [23]. For these patients, we used several teleconferencing applications including Microsoft Teams, Microsoft Skype, and Cisco’s WebEx to administer surveys and conduct HUAs.

We used The Gambler II to perform health utility assessments [11]. For survey data collection, we used REDCap, a web-based survey platform designed to capture users’ data with web forms [18]. All data were collected and stored on a secure server at the University of Cincinnati. Access to data was restricted to institutional review board–authorized users with login credentials. We analyzed data using Python (Python Software Foundation) and R (The R foundation) [24-26].

## Results

### Demographic Data

We recruited 71 patients from 4 DCI sites in the Cincinnati Metropolitan area. A total of 63 patients consented and completed the study. Of those who consented, 62 (98%) enrolled in the study during dialysis clinic visits, while 1 (2%) enrolled through teleconferencing software at their residence. The cohort’s age range was from 24 to 80 years, with a median age of 59 years and mean age of 58 years (Multimedia Appendix 1). Moreover, 44 (70%) patients had African American racial backgrounds, and 19 (30%) were European American. A high school diploma was the most frequent highest level of educational attainment (Table 1). The median Rapid Estimate of Adult Literacy in Medicine Short Form and subjective numeracy scores were 7.0 and 4.0, respectively. In addition, of the 63 patients, 54 (86%) had not received a previous kidney transplant, and 47 (75%) had an interest in receiving a kidney transplant. The participants spent an average of 5.9 years on dialysis (African Americans: 7.3 years, European Americans: 2.79 years; *P*=.07; Multimedia Appendix 1).

**Table 1:** Summary statistics of the population broken into race-matched versus race-mismatched study arms.

Characteristics		Values			
		Overall (n=63)	Race-matched videos (n=33)	Race-mismatched videos (n=33)	*P* value
Age (years), mean (SD)		57.8 (12.3)	56.1 (13.5)	59.8 (10.8)	.26
**Race or ethnicity, n (%)**					.80
	African American	44 (69.8)	24 (72.7)	20 (66.7)	
	European American	19 (30.2)	9 (27.3)	10 (33.3)	
**Gender, n (%)**					.54
	Female	30 (47.6)	14 (42.4)	16 (53.3)	
	Male	33 (52.4)	19 (57.6)	14 (46.7)	
**Highest education level attained, n (%)**					.57
	Less than high school diploma	10 (15.9)	4 (12.1)	6 (20.0)	
	High school or general education diploma	25 (39.7)	16 (48.5)	9 (30.0)	
	Some college, no degree	11 (17.5)	6 (18.2)	5 (16.7)	
	Associate degree	5 (7.9)	3 (9.1)	2 (6.7)	
	Bachelor’s degree	9 (14.3)	3 (9.1)	6 (20.0)	
	Master's degree	3 (4.8)	1 (3.0)	2 (6.7)	
Years on dialysis, mean (SD)		5.9 (8.1)	6.79 (7.8)	5.0 (8.3)	.07
History of previous kidney transplant, n (%)		9 (14.3)	6 (18.2)	3 (10.0)	.48
**Interested in receiving a kidney transplant, n (%)**					.64
	Maybe	3 (4.8)	1 (3.0)	2 (6.7)	
	No	13 (20.6)	8 (24.2)	5 (16.7)	
	Yes	47 (74.6)	24 (72.7)	23 (76.7)	

### Knowledge Assessments

We conducted a knowledge assessment using a 10-item questionnaire administered before the utility assessment as a pretest and after as a posttest (Multimedia Appendix 1). The Cronbach alpha for the pretest was .990, and it was .994 for the posttest. As shown in Tables 2 and 3, for the cohort, the improvement in test scores (mean and median 10.0 points) following the utility assessment process and viewing of health state videos were clinically and statistically significant (*P*<.001, paired Wilcoxon *t* test). However, there was not a statistically significant difference in pretest versus posttest change scores between the 2 study arms, or between African Americans compared with European Americans, with *P* values of .95 and .96 (Mann-Whitney test), respectively.

**Table 2:** Evaluation of patients’ health literacy, numeracy, and their knowledge of end-stage kidney disease and hepatitis C stratified by study arm.

Tests	Overall, mean (SD)	Race-matched videos, mean (SD)	Raced-mismatched videos, mean (SD)	*P* value^a^
Before test	79.0 (17.3)	80.2 (17.2)	77.7 (17.7)	.59
After test	89.0 (15.9)	89.6 (15.8)	88.2 (16.1)	.77
Numeracy	3.9 (1.1)	4.1 (1.0)	3.7 (1.1)	.14
REALM-SF^b^	6.1 (1.6)	6.1 (1.5)	6.1 (1.8)	.98
Change score	10.0 (13.8)	9.5 (12.7)	10.5 (15.0)	.09

^a^*P* value denotes comparison between race-matched and race-mismatched population health utilities. This assumes that if *P*≤.05, there was a significant difference in health utilities between race-matched (same race) and race-mismatched (different race) health utilities.

^b^REALM-SF: Rapid Estimate of Adult Literacy in Medicine Short Form.

**Table 3:** Evaluation of patients’ health literacy, numeracy, and their knowledge of end-stage kidney disease and hepatitis C stratified by race.

Tests	Overall, mean (SD)	African American, mean (SD)	European American, mean (SD)	*P* value^a^
Before test	79.0 (17.3)	78.4 (16.0)	80.4 (20.5)	.42
After test	89.0 (15.9)	88.3 (15.6)	90.4 (16.7)	.45
Numeracy	3.9 (17.3)	3.8 (1.1)	4.2 (1.0)	.18
REALM-SF^b^	6.1 (1.1)	5.9 (1.9)	6.6 (0.77)	.26
Change score	10.0 (13.8)	9.9 (13.2)	10.1 (15.4)	.96

^a^*P* value denotes comparison between African and European American population health utilities. This assumes that if *P*≤.05, there was a significant difference in health utilities between European and African American health utilities.

^b^REALM-SF: Rapid Estimate of Adult Literacy in Medicine Short Form.

### End-Stage Kidney Disease Health Utilities

**Figure 4:**
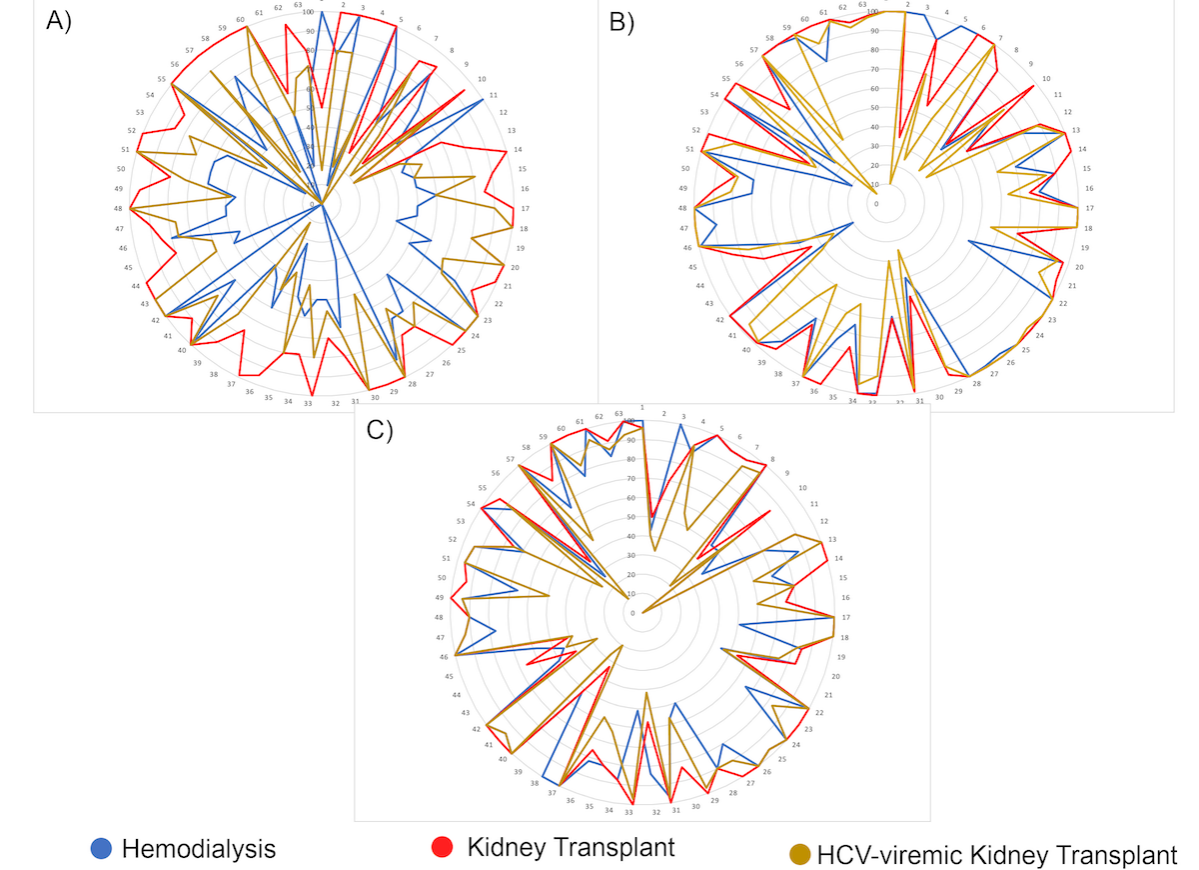
Radar plot of the health state utilities. (A) Visual analog scale; (B) standard gamble; and (C) time trade-off. The 3 figure panels show the utilities assessed for each of the 63 patients in the study. Each panel summarizes results for the 3 different utility assessment methods. Different colors are used to represent each of the 3 health states: hemodialysis, transplantation with a hepatitis C (HCV)–unexposed kidney, and transplantation with an HCV-viremic kidney. Each number on the outside circle represents a single patient’s utility scores.

We assessed utilities from patients on chronic intermittent hemodialysis for 3 health states relevant to decision-making about kidney transplantation. The results are reported in Tables 4 and 5 and are depicted pictorially in Figure 4. With all 3 assessment methods, means utilities were highest for transplantation with an HCV-unexposed kidney. Hemodialysis had the lowest average utility assessed with the VAS, while transplantation with an HCV-viremic kidney had the lowest utility using the TTO and SG. Looking at ranking of utilities within each patient, 47 (75%) and 39 (62%) of the patients rated transplantation with an HCV-viremic kidney lower than hemodialysis with SG utilities and TTO utilities, respectively.

**Table 4:** Health utilities evaluation of race-matched vs race-mismatched video patient cohort.

Health utilities		Overall, mean (SD)	Race-matched videos, mean (SD)	Race-mismatched videos, mean (SD)	*P* value^a^
**Visual analog scale**					
	Hemodialysis	57.9 (25.9)	63.2 (26.7)	52.0 (25.3)	.11
	Kidney transplant	88.2 (17.8)	85.2 (22.3)	91.5 (10.2)	.68
	Hepatitis C–viremic kidney transplant^b^	66.30 (27.3)	65.6 (27.4)	67.1 (27.7)	.94
**Standard gamble**					
	Hemodialysis	82.5 (23.1)	85.3 (20.5)	79.4 (25.6)	.26
	Kidney transplant	89.0 (18.0)	87.4 (19.4)	90.7 (15.8)	.67
	Hepatitis C–viremic kidney transplant^b^	75.5 (28.2)	75.5 (29.7)	75.5 (26.9)	.68
**Time trade-off**					
	Hemodialysis	80.3 (20.5)	78.2 (21.0)	82.6 (20.0)	.39
	Kidney transplant	84.8 (22.0)	85.4 (22.6)	84.2 (21.7)	.74
	Hepatitis C-viremic kidney transplant^b^	73.8 (28.1)	75.1 (27.0)	72.3 (29.6)	.76

^a^*P* value denotes comparison between race-matched and race-mismatch population health utilities. This assumes that if *P*≤.05, there was a significant difference in health utilities between race-matched (same race) and race-mismatched (different race) health utilities.

^b^Health utility normalization equation: Raw hepatitis C virus utility * Kidney transplant utility = hepatitis C virus utility.

**Table 5:** Health utilities evaluation of the African American versus European American patient cohort.

Health utilities		Overall, mean (SD)	African American, mean (SD)	European American, mean (SD)	*P* value^a^
**Visual analog scale, mean (SD)**					
	Hemodialysis	57.9 (25.9)	59.1 (27.4)	55.1 (22.4)	.38
	Kidney transplant	88.2 (17.8)	86.7 (20.3)	91.8 (9.3)	.73
	Hepatitis C–viremic kidney transplant^b^	66.30 (27.3)	65.6 (27.4)	68.0 (27.8)	.70
**Standard gamble, mean (SD)**					
	Hemodialysis	82.5 (23.1)	83.0 (25.2)	81.3 (17.6)	.22
	Kidney transplant	89.0 (18.0)	88.8 (19.6)	89.4 (14.3)	.52
	Hepatitis C–viremic kidney transplant^b^	75.5 (28.2)	77.5 (28.5)	70.9 (27.5)	.24
**Time trade-off**					
	Hemodialysis, mean (IQR; SD)	80.3 (66.0-100.0; 20.5)	83.2 (75.0-100.0; 21.0)	73.6 (66.0-83.5; 18.2)	.04
	Kidney transplant, mean (SD)	84.8 (22.0)	85.2 (23.8)	84.0 (17.7	.16
	Hepatitis C–viremic kidney transplant^b^, mean (SD)	73.8 (28.1)	75.4 (29.7)	70.0 (21.0)	.20

^a^*P* value denotes comparison between race-matched and race-mismatch population health utilities. This assumes that if *P*≤.05, there was a significant difference in health utilities between race-matched (same race) and race-mismatched (different race) health utilities.

^b^Health utility normalization equation: Raw hepatitis C virus utility * Kidney transplant utility = hepatitis C virus utility.

Utility assessments for transplantation with an HCV-infected kidney were carried out with anchor states of transplant with HCV-unexposed kidney as the best outcome and death as the worst outcome. We normalized these utilities to the same 0-100 scale used for the other health states by multiplying the raw utility for transplant with HCV-exposed kidney times the utility of transplant with an HCV-unexposed kidney. Normalized utility means using VAS, SG, and TTO assessments were 66.3, 75.5, and 73.8, respectively. Looking at the raw SG utility weights for transplantation with an HCV-viremic kidney, given a choice between transplantation with an HCV-unexposed kidney and an HCV-viremic kidney, patients were willing to take a 24.5% chance of dying to avoid receiving an HCV-viremic kidney. We did not find statistically significant differences in utilities between race-matched and race-mismatched study arms. However, we did find that African Americans on average had higher utility weights than European Americans for hemodialysis evaluated with the TTO (83.2 versus 73.6, respectively; *P*=.04 [Mann-Whitney]).

## Discussion

### Principal Results

Regarding our primary study goal, we found that using our health utility assessment tool in an ambulatory community dialysis clinic setting was feasible. The entire process took approximately 1 hour per patient, with health utility assessments taking approximately 30 minutes on average. Barriers regarding home internet access may have increased the acceptability of utility assessment in the community dialysis clinic setting [27]. Many patients expressed pleasure at the opportunity to engage in a value-added activity while they were “captive” during their 3- to 4-hour dialysis session. This bodes well for plans to conduct shared decision-making visits using The Gambler II platform in these same community dialysis clinics.

### Secondary Results

Our second goal was to collect health utilities from patients with dialysis for several ESKD health states relevant to the decision about kidney transplantation. We found a wide variation in health utilities from patient to patient. Consistent with prior studies, utilities assessed using the SG technique were higher than those determined with the VAS or TTO techniques. SG holistically incorporates risk attitude into its assessment, and since most people are risk averse, SG utilities tend to be higher [28]. Of note, there was a wide variation in utilities for transplantation with an HCV-viremic kidney, and in a few instances, patients had lower utilities for this health state than for continued hemodialysis. While mean SG utilities for transplant with an HCV-viremic kidney were lower than hemodialysis (75.5 vs 82.5), standard deviations were large (28 and 23, respectively), which further demonstrated significant differences in utility values across the patients. To this point, 30 (48%) patients had higher SG utilities for transplant with an HCV-viremic kidney compared with hemodialysis. We also explored whether there were racial differences in health state utilities between patients with African American and European American backgrounds. We found African Americans had higher TTO utilities for hemodialysis compared to their European American counterparts. One possible explanation is the phenomenon of accommodation. While quality of life may diminish markedly when patients move from a better state of health to a health state marred by chronic disease or disability, studies have shown that, over time, many patients accommodate to the new health state with an accompanying improvement in assessments of quality of life [29,30]. Indeed, in subanalyses stratified by race, African Americans had a significantly longer dialysis vintage (7.3 years) compared with European Americans (2.8 years; *P*=.04).

During the testing of The Gambler II in a clinical setting, the COVID-19 pandemic interrupted patient recruitment. We performed a subanalysis on patients who enrolled after the COVID-19 interruption. We found that gender and study arm did not have a statistically significant impact on knowledge scores or utilities. Stratifying by race, we found that African Americans had higher SG utilities for all but hemodialysis (Multimedia Appendix 1). Furthermore, African Americans had higher TTO utilities for all 3 health states (Multimedia Appendix 1). African Americans had a mean utility of 87.6 for transplantation with an HCV-viremic kidney compared to a mean utility of 69.3 for European Americans (*P*=.004) during the pandemic. We did not see similar differences among patients in the prepandemic cohort given the small sample size of European Americas (n≤5).

To our knowledge, this is the first paper directly eliciting health utilities from patients with dialysis for transplant with an HCV-viremic kidney. Our major finding is that patients’ utilities for this outcome vary dramatically, and that for some patients, the worry and concern associated with even a successful transplant of an HCV-viremic kidney may result in a utility weight lower than their current health state of chronic intermittent hemodialysis. We found a slight negative correlation between the difference between the SG utility for transplant with an HCV-viremic kidney and hemodialysis and dialysis vintage. For patients who have a longer dialysis vintage, SG utility for transplant with an HCV-viremic kidney and hemodialysis had a negative correlation (–0.16). This means that there is a trend toward a more negative view of transplant with an HCV-viremic kidney compared with hemodialysis among patients who have been on dialysis for a longer time. We also examined how patients’ history of a failed kidney transplant may affect health utilities. We compared this patient population hemodialysis and transplant with an HCV-viremic kidney SG utilities. There was a compelling but not statistically significant trend toward a larger decrement in this value among patients with prior failed kidney transplant (–15.6 versus –5.5; *P*=.29). However, our cohort only had 9 (14%) patients with a prior transplant.

Regarding our third goal, our study showed that health state videos narrated by patient actors, viewed as part of the process of assessing patients’ values and preferences for health outcomes of ESKD, can improve knowledge about these health states. On average, knowledge scores improved by 10 points between the pretest and posttest, demonstrating that these videos can educate patients about the relevant health states as a beneficial side effect of the utility assessment process. While we expected to find a positive impact of using race-matched exemplars compared with race-mismatched exemplars, there were no significant differences between study arms on knowledge gain. Whether our study simply lacked the power to detect a difference and whether matching patient demographics truly matters is a question for further investigation. Of note, patients watching videos narrated by African American patient actors had greater knowledge gains (median 10.0) than those viewing videos narrated by European American patient actors (median 5.0), although this was not statistically significant (*P*=.25).

Regardless, patients found the videos informative and impactful. Following the utility assessment process, several patients voiced their appreciation for the video descriptions of health states. Some patients even reported being moved to tears.

### Comparison With Prior Work

Prior studies noted the potential benefits of using HCV-viremic kidneys to expand the pool of organs available to hemodialysis patients, thus reducing waiting times for transplantation and providing opportunities for transplantation in patients who might not have received a kidney otherwise. Cost-effectiveness analyses have shown the strategy to be cost-effective at a policy level for the general population of patients with ESKD [13,22]. However, none of these analyses used utility assessments from actual patients for the outcome transplant with an HCV-viremic kidney [31,32]. Given the marked patient-to-patient variability in utilities for the 3 health states we studied, patient preferences for health states must be considered in the shared decision-making process about transplantation with an HCV-viremic versus an HCV-uninfected kidney.

The Gambler II provides a consistent and efficient platform to elicit patient utilities and could be integrated into a tool to facilitate shared decision-making. One could envision such a tool that performs personalized decision analyses, using a combination of individual patient’s utilities along with clinical and demographic information needed to estimate organ waiting list times, to provide estimates of quality-adjusted survival or life expectancy with each strategy for that individual patient.

### Limitations

Our study had some limitations. Given the demographics of the Cincinnati metropolitan area dialysis clinics in our study, it was difficult to recruit patients with European American racial backgrounds in equal numbers to those with African American backgrounds. In addition, the COVID-19 pandemic negatively impacted our total recruitment due to a pause in study activities between March and October of 2020 [23]. A possible contributing factor to utility weights for transplant with an HCV-viremic kidney being lower than hemodialysis for some patients was the way we assessed utilities for this health state separately from hemodialysis and transplant with an HCV-unexposed kidney. We wanted an explicit measure of the utility differences between transplant with an HCV-unexposed and an HCV-viremic kidney. This measure would include in the SG assessment an estimate of the willingness of patients to risk death to avoid such a transplant. However, the normalized utility values for this health state were not directly assessed compared with transplant with an HCV-unexposed kidney.

### Conclusions

In conclusion, we found that it is feasible to use a computer platform to assess patients’ utilities for health states related to ESKD in ambulatory community dialysis clinics. Furthermore, we demonstrated that utility weights vary dramatically from person to person. These findings have implications for the future development of shared decision-making tools to aid clinicians and their patients with the challenging question of whether to accept transplantation with HCV-viremic kidneys in patients to reduce waiting times and decrease time spent on hemodialysis. This decision will depend upon both expected organ–waiting list times for HCV-unexposed and HCV-exposed kidneys and individual patient’s values and preferences for these relevant health states.

## Appendix

Multimedia Appendix 1 Manuscript appendix containing select demographic information about the patients, the questions asked of patients before and after the assessment, and information given to patients throughout the assessment. It also contains the explanation for the health utility assessments, screenshots of the assessments, and utility information generated from the patient assessment.

## Abbreviations

DCI: Dialysis Center Inc
ESKD: end-stage kidney disease
HCV: hepatitis C virus
HUA: health utility assessment
REDCap: Research Electronic Data Capture
SDM: shared decision-making
SG: standard gamble
TTO: time trade-off
VAS: visual analog scale

## Data Availability

Data related to this study and The Gambler software are available to use upon request.
