# Supplementary material for: Implementing a Health Utility Assessment Platform to Acquire Health Utilities in a Hemodialysis Outpatient Setting: Feasibility Study
Source: JMIR Form Res. 2022 Jul 28;6(7):e33562. doi: 10.2196/33562 (PMC9377480; doi:10.2196/33562)
Supplement: Multimedia Appendix 1 [file formative_v6i7e33562_app1.docx]

**Appendix A: Health utility assessment methods**

Health utilities are quality of life weighing factors assessed on a health state acquired via a health utility assessment.

Visual Analog Scale: The health state gets evaluated on scale from 0 (worst health state) to 100 (best health state). The assessor will place the health state in between these two anchor values on the to obtain the value.

Standard Gamble: Two options will present themselves: the confirmed intermediate health state as the first option or taking the gamble as the second option. The gamble, if chosen has a patient draw a pill from a pill bottom at random with a certain percent chance of the pill curing landing them on the best health state (cure pill) and the possibility of the pill landing on the worst health state (poison pill). The equation to obtain the health utility, once the patient cannot make a decision on either option, is the following: Utility = 1 – (Number of poison pills / Total Number of pills in the bottle)

Time tradeoff: The time tradeoff accounts for a person’s life expectancy to present different possibilities of health state valuation. One possibility is to be in the intermediate health state for the person’s full life expectancy. The other possibility assumes that for being the worst health state for a certain time period (often times passing away early) the patient will be in the best health state for a certain time period. The equation for the utility acquired once a patient feels ambivalent to the choices is the following: Utility = (Time in Best Health State) / (Total Life Expectancy)

**Appendix B – Knowledge Survey**

1. What is/are the most common cause(s) of End Stage Kidney Disease?
   1. Cancer
   2. **Diabetes**
   3. **High Blood Pressure**
   4. Stroke
2. What is a purpose of the kidney?
   1. **Filter the blood and remove waste from the body**
   2. Aid in moving blood throughout the body
   3. **Help maintain salt and water balance**
   4. Act as a filter for lymph in the body
3. What complication(s) can happen if a person does not treat Hepatitis C?
   1. Angina
   2. Blurry vision
   3. **Liver damage**
   4. Occasional migraines throughout the day
4. What some steps that must happen for you to receive a kidney transplant?
   1. **Be placed on a waiting list for a kidney**
   2. **Have your blood type checked**
   3. **Availability of a kidney from a donor**
   4. **Have surgery to implant the new kidney**
5. What is the purpose of taking medication after a kidney transplant?
   1. To prevent allergic reactions
   2. To prevent the eyes from yellowing
   3. To improve ease of breathing
   4. **To prevent your body from rejecting the kidney**
6. What kind of medications would someone have to take as a result of being transplanted with a hepatitis C-infected kidney?
   1. Blood pressure medications
   2. Antihistamines for allergic reactions
   3. **Antiviral medications to eliminate infection with the hepatitis C virus**
   4. Antibiotics
7. How long is the average treatment course for hepatitis C?
   1. 6 weeks
   2. 8 weeks
   3. **12 weeks**
   4. 24 weeks
8. What are some of the possible advantages to accepting transplantation with a kidney from a hepatitis C-infected donor?
   1. **Hepatitis C-infected donors frequently are younger and have fewer medical conditions that might hurt the quality of the donor kidney**
   2. You don’t need to take medications to prevent organ rejection
   3. **Possibly shorter time waiting to receive a donor kidney**
   4. Receiving a hepatitis C-infected kidney will make you immune to hepatitis C
9. What are some of the concerns associated with getting transplanted with a hepatitis C-infected kidney?
   1. You might develop type II diabetes
   2. **You might get infected with hepatitis C**
   3. If you have hypertension, it will get worse
   4. **You will have to take additional medications to treat a hepatitis C infection**
10. What are some signs and symptoms of kidney failure?
    1. **Your legs swell up**
    2. **You may not have to pee as often**
    3. **You feel tired all the time**
    4. The whites of your eyes turn yellow

Note: the correct answers in the survey above are **bolded**.

**Appendix C. Randomization technique for entering patients in race matched or race mismatched video arm**

A team member (HJD) prepared a set of 90 randomization envelopes and recused themselves from the consenting and retrieving of the randomization codes. The slips were marked either Same Race (SR), representing the race-matched health state video arm or Different Race (DR), representing the race-mismatched health state video arm. This set of 90 had randomization blocks of 6, to SR or DR with 3 SR and 3 DR in each block. They placed a code slip inside each numbered envelope according to the randomization sequence.

There were 51 AA subject envelopes and 39 non-AA subject envelopes. AAA opened each envelope sequentially with one exception at the time consent was obtained. This exception was due to human error.

**Appendix D. Health State Descriptions and Video Clip Scripts**

***Brief Descriptions of Intermediate Health States -***

**
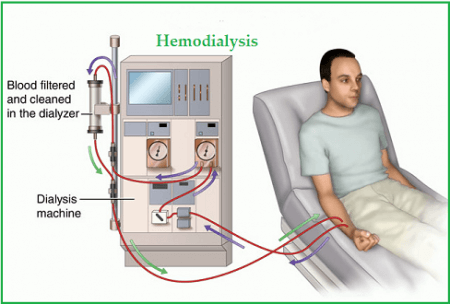
Hemodialysis**:

You are very familiar with this health state since you have end stage kidney disease and are currently receiving hemodialysis. As you know, your kidneys do not work, and you must come to a community-based center 3 days each week for a 3 – 4-hour dialysis session. You have either a fistula or catheter in place to allow vascular access for dialysis. Blood work is checked frequently. It is challenging to leave town for travel or vacation, as you must be sure to have arrangements for dialysis wherever you may go.


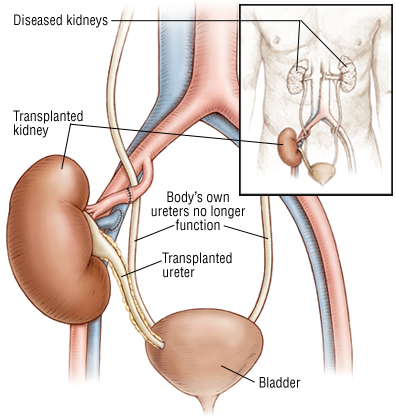


**Kidney transplant**:

You have been fortunate enough to receive a kidney from a deceased donor. You had to wait a long time to get this kidney, but it finally happened. You no longer need to have dialysis. You have regained a lot of freedom, and don’t need to be seeing your kidney doctors or nurses as often as when you were on dialysis. You do need to take medications to prevent rejection of your transplanted kidney.

**
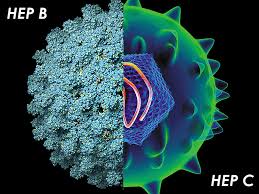
Kidney transplant from donor infected with hepatitis C**:

You decided it was worth accepting a kidney from a deceased donor who was infected with hepatitis C. As a result, you received your kidney faster than if you had waited for a non-hepatitis C infected kidney. You did worry a little about what the hepatitis C infection might do to your liver. You had to take medicines for 12 weeks to treat the hepatitis C infection you got from the transplant, but now it has been cured. You no longer need to have dialysis. You have regained a lot of freedom, and don’t need to be seeing your kidney doctors or nurses as often as when you were on dialysis. You do need to take medications to prevent rejection of your transplanted kidney.

***Standardized Scripts for Patient Actors Portraying Health States in Videos -***

**Hemodialysis**:

Hello, my name is ____. I have end stage kidney disease and have been on dialysis for 5 years. I have thought about kidney transplantation, but I am not sure that will ever happen. Before I started my treatment, I used to find it hard to concentrate. I wouldn’t use the restroom as often, and my legs would swell, feeling like they had water in them. One day, I went to the doctor and told her about my symptoms. She did some tests and found out that my kidneys were failing. She said that I would need to be on a dialysis machine to replace what my kidneys did to remove bad stuff from my blood. The dialysis machine takes blood out of my body, cleans it, and then gives the blood back to me. In order to do that, I needed to have a catheter placed, until a fistula was ready to use. They now hook me up to the fistula for my dialysis sessions. This means I get needles stuck in my arm every time I get dialysis. I have to come to a dialysis center three times a week, which makes it hard to do much anything else. I have to spend 3 or 4 hours in a chair while I have dialysis. I usually just sleep to pass the time. Sometimes it is hard for me to get to the dialysis center, and I worry about that too. Although it was scary at first, I’ve gotten used to living this way.

**Kidney Transplant**:

Hello, my name is ___. I had end stage kidney disease and was receiving dialysis for 5 years. I recently had surgery for a kidney transplant. I had to wait a long time, about four years before a donor kidney became available. While waiting, I was on dialysis, going to the clinic three times a week. I had to deal with a fistula and getting needles stuck in my arm for every session. A few months ago, my doctors contacted me to say they had found a kidney donor that was a match for me. I had the transplant done, and now my life has changed. I have a new kidney that works and I don’t have to go to dialysis any more. I feel free! I don’t have to see my kidney doctors or nurses anywhere near as often as I used to. I can leave town to visit friends and family, and don’t have to worry about when my next dialysis session is. I do have to take new medicines to prevent rejection of my kidney. I do worry about that a little, rejection that is. But, all in all, my life is much better!

**Kidney transplant from donor infected with hepatitis C**:

Hello, my name is ___. I had end stage kidney disease and was receiving dialysis for about 3 years. I recently had surgery for a kidney transplant. Because I was willing to accept a kidney from a donor who was hepatitis C positive, I was able to get a kidney sooner than I might have otherwise. I was on the waiting list for about two years before a such a donor kidney became available. While waiting, I was on dialysis, going to the clinic three times a week. I had to deal with a fistula and getting needles stuck in my arm for every session. A few months ago, my doctors contacted me to say they had found a kidney donor that was a match for me. I had the transplant done, and now my life has changed. I have a new kidney that works and I don’t have to go to dialysis any more. I feel free!

Because I received a kidney from a donor with hepatitis C, I had to get treated with antivirus medicines for 12 weeks. I was kind-of worried about this, since there was a small chance the antivirus medicines might not work. If that happened, I could have had liver damage. But I am happy to say that my antivirus treatment is finished and my doctors tell me I am no longer infected with the hepatitis C virus. Now, I don’t have to see my kidney doctors or nurses anywhere near as often as I used to. I can leave town to visit friends and family, and don’t have to worry about when my next dialysis session is. I do have to take new medicines to prevent rejection of my kidney. I do worry about that a little, rejection that is. But, all in all, my life is much better!

**Appendix E: Select Demographic information stratified by race**

|  | Overall | African American | European American | P-Value |
| --- | --- | --- | --- | --- |
| n | 63 | 44 | 19 |  |
| age, mean ± SD | 57.8 ± 12.3 | 57.36 ± 11.6 | 58.95 ±14.2 | 0.36 |
| Years on dialysis, mean ± SD | 5.9 ± 8.1 | 7.30 ± 9.2 | 2.79 ± 3.0 | 0.07 |

**Appendix F: Utilities for transplantation with HCV-viremic kidney with anchor states of Transplantation with HCV-unexposed kidney and Dead.**

|  | Overall | Race Matched  Videos | Race Mismatched  Videos | P-Value |
| --- | --- | --- | --- | --- |
| Visual Analog Scale (VAS) | | | | |
| Hepatitis C-viremic Kidney Transplant, mean ±SD | 74.3  ±26.2 | 75.4  ±25.9 | 73.2  ±28.1 | 0.922 |
| Standard Gamble (SG) | | | | |
| Hepatitis C-viremic Kidney Transplant, mean ±SD | 82.4  ±22.5 | 82.5  ±22.2 | 82.2  ±23.2 | 0.718 |
| Time Trade-off | | | | |
| Hepatitis C-viremic Kidney Transplant, mean ±SD | 83.5  ±22.4 | 83.8  ±22.8 | 83.0  ±22.3 | 0.81 |

We assessed Transplantation with an HCV-viremic Kidney using anchor states of Transplantation with an HCV-unexposed Kidney and Death. We normalized these results on a zero to one-hundred scale as follows: Raw HCV-viremic Kidney Transplant utility * HCV-unexposed Kidney Transplant Utility = HCV-viremic Kidney Transplant normalized utility.

**Appendix G. Raw health state utilities by race.**

|  | Overall | African American | European American | P-Value |
| --- | --- | --- | --- | --- |
| Visual Analog Scale (VAS) | | | | |
| Hepatitis C-viremic Kidney Transplant, mean ±SD | 74.3  ±26.2 | 74.3  ±25.4 | 74.5  ± 28.7 | 0.844 |
| Standard Gamble (SG) | | | | |
| Hepatitis C-viremic Kidney Transplant, mean ±SD | 82.4  ±22.5 | 84.3  ± 20.5 | 78.0  ± 26.6 | 0.3 |
| Time Trade-off | | | | |
| Hepatitis C-viremic Kidney Transplant, median (mean) ±SD | 83.5  ±22.4 | 84.3  ± 23.1 | 81.6  ± 21.0 | 0.494 |
|  | | | | |

**Appendix H. Health Utilities stratified by race post-COVID 19 shutdown.**

|  | Overall | African American | European American | P-Value |
| --- | --- | --- | --- | --- |
|  | 41 | 25 | 16 |  |
| Visual Analog Scale (VAS) | | | | |
| Hemodialysis, mean ± SD | 54.5 ±22.9 | 54.4 ±25.0 | 54.7 ±19.8 | 0.723 |
| Kidney Transplant, mean ± SD | 91.2 ±10.8 | 91.4 ±11.5 | 90.9 ±9.9 | 0.753 |
| Hepatitis C-viremic Kidney Transplant, mean ± SD † | 72.1 ±23.4 | 75.2 ±19.8 | 67.3 ±28.2 | 0.563 |
| Hepatitis C-viremic Kidney Transplant, mean ± SD Raw Utility | 79.2 ±22.7 | 82.0 ±17.4 | 74.7 ±29.4 | 0.615 |
| Standard Gamble (SG) | | | | |
| Hemodialysis, mean ± SD | 82.7 ±22.9 | 83.6 ±25.7 | 81.2 ±18.3 | 0.206 |
| Kidney Transplant, mean ± SD | 92.3 ±14.3 | 95.3 ±13.3 | 87.6 ±14.9 | 0.023 |
| Hepatitis C-viremic Kidney Transplant, mean ± SD † | 82.8 ±24.6 | 89.2 ±19.4 | 72.9 ±28.8 | 0.044 |
| Hepatitis C-viremic Kidney Transplant, mean ± SD Raw Utility | 88.3 ±20.4 | 92.8 ±13.6 | 81.2 ±27.0 | 0.124 |
| Time Trade-off | | | | |
| Hemodialysis, mean ± SD | 80.6 ±18.8 | 85.5 ±18.1 | 72.8 ±17.6 | 0.017 |
| Kidney Transplant, mean ± SD | 89.8 ±16.1 | 93.9 ±12.8 | 83.4 ±18.8 | 0.006 |
| Hepatitis C-viremic Kidney Transplant, mean ± SD † | 80.5 ±23.6 | 87.6 ±20.0 | 69.3 ±25.0 | 0.004 |
| Hepatitis C-viremic Kidney Transplant, mean ± SD Raw Utility | 88.4 ±19.2 | 93.0 ±15.6 | 81.3 ±22.3 | 0.057 |
| † Health utility normalization equation: Raw HCV utility* Kidney Transplant Utility = HCV utility | | | | |


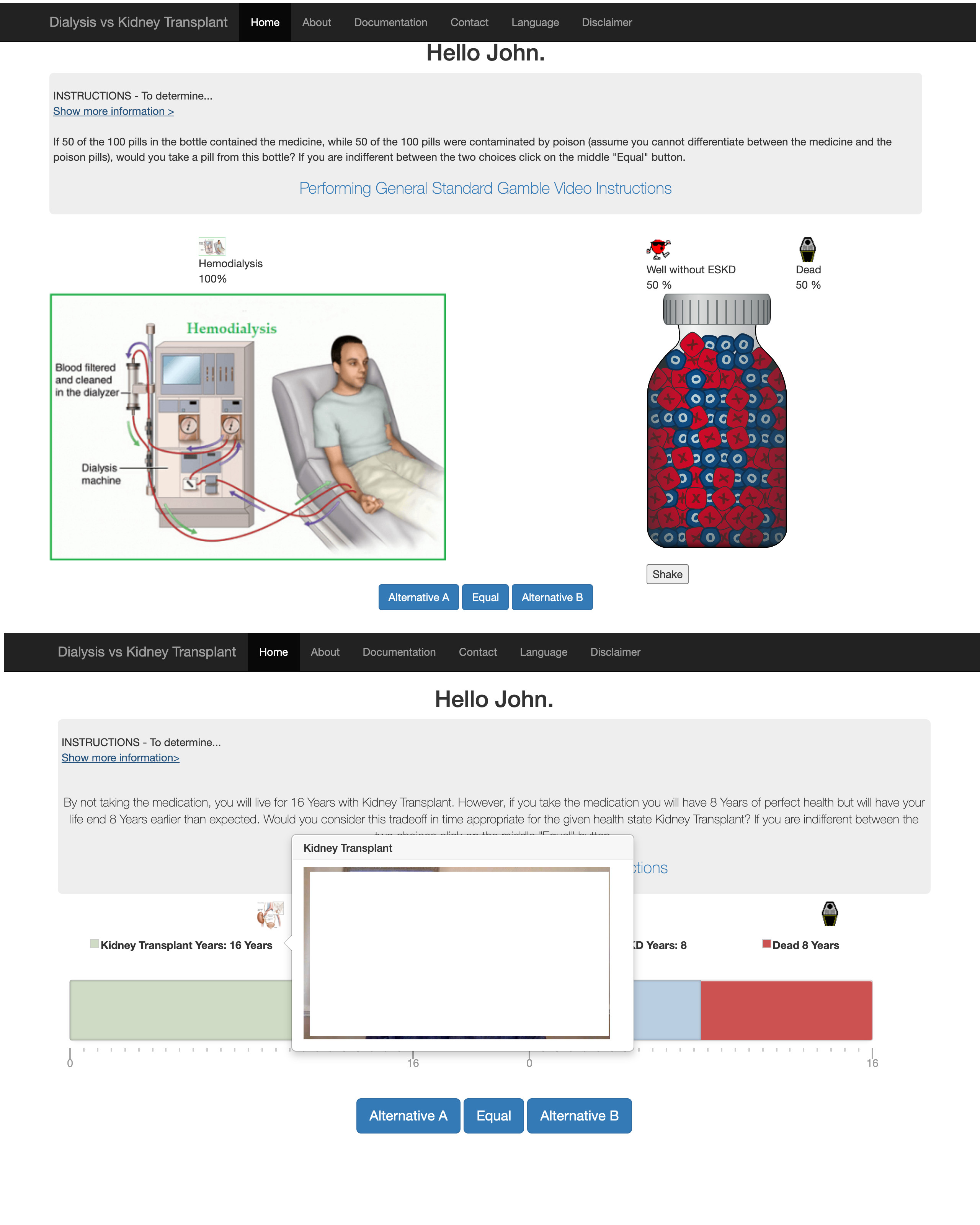
Supplemental Figure 1. Screenshots of different health utility assessments. Top: Patient evaluating the hemodialysis health state through the standard gamble utility assessment. Patients repeatedly make choices between hemodialysis, the health state being assessed, or accepting the gamble between cure and death, by choosing the pill bottle representing the gamble. In the figure above, the pill bottle contains 50 cure and 50 poison pills, representing a 50:50 gamble on cure vs death. Bottom: Patients evaluating kidney transplant using the time trade-off, The Gambler uses life tables to determine the duration of life expectancy for the time trade-off. In this example, patients are asked to choose between living with a kidney transplant for 16 years or passing away 8 years earlier in exchange for living a shorter time (8 years) in a better state of health, Well without End Stage Kidney disease. The video clip in the figure demonstrates how a user can watch a demographically-matched patient actor describes the health state being assessed (face hidden for privacy concerns).


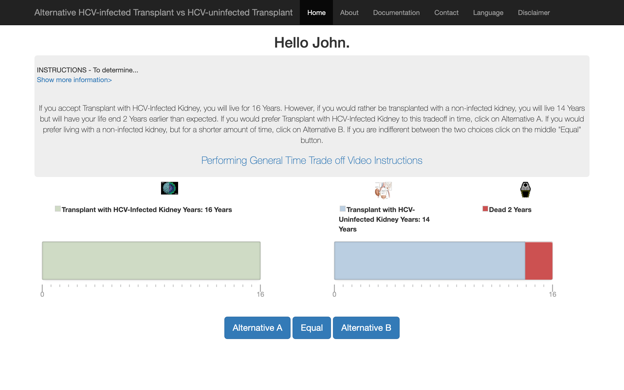
Supplemental Figure 2. Screenshot of patient going through the time trade-off health utility assessment for Transplantation with an HCV-Infected Kidney. This assessment differs from others in that the best anchor health state is Transplantation with an Uninfected Kidney.
